# Cleanliness a Preservative of the Teeth

**Published:** 1860-05

**Authors:** 


### CLEANLINESS A PRESERVATIVE OF THE TEETH.

(Prepared expressly for popular reading and for reprint in the newspaper press.)

We *wash* our *hands* and *face*. We keep, or should keep our entire body clean, as cleanliness is conducive to health. We are always careful to wash our hands before eating, and are somewhat particular as to the cleanliness of our food. Now while careful in these particulars, which is all very proper, how strange it is that we should neglect the far more important matter—important in a two-fold sense—the cleanliness of the *mouth*, for it has more to do with the food than the hands, and its secretions are necessary to the proper preparation of the food for the stomach. Now a *foul* mouth will necessarily vitiate the secretions, and which being mingled with the food are carried into the stomach, resulting in impaired digestion.

This will surely strike every reader as sufficiently offensive to induce its correction.

But there is another equally important reason for a clean mouth, viz.:—the *Preservation of the teeth.*

By not keeping the teeth cleaned, food—both animal and vegetable—is allowed to remain in the spaces between the teeth which by the warmth of the mouth, becomes putrid, and is supposed to form an acid that acts upon the enamel of the teeth thus producing decay, which is attended with pain, and the subsequent loss of these important organs.

It is necessary the teeth be cleaned at least twice a day—before going to bed and on rising in the morning—and that *each tooth be thoroughly cleaned*, and the surfaces where the brush will not touch may be reached with floss silk waxed with beeswax, and passed back and forth between the teeth. Where the silk can not be obtained, substitute a quill toothpick, made thin enough to pass freely between them and by which all the food or other matter deposited there, shall be entirely removed.

Cleanliness of the mouth, therefore, is conducive to the general health as well as the preservation of the teeth.

PHILADEPHIA, 1860. O. U. C.

